# Supplementary material for: Collaborative intelligence in AI: Evaluating the performance of a council of AIs on the USMLE
Source: PLOS Digit Health. 2025 Oct 9;4(10):e0000787. doi: 10.1371/journal.pdig.0000787 (PMC12510544; doi:10.1371/journal.pdig.0000787)
Supplement: S2 File — (ZIP) [file pdig.0000787.s002.zip › S2 Data Raw data files containing unprocessed question inputs and LLM outputs including transcripts of deliberations/STEP3_Question-45-1_Type-MC-NJ.docx]

**Facilitator** **[Round 1]:** A 22-year-old man comes to the emergency department because of a 4‐day history of cough productive of green sputum as well as fever, chills, and rigors. He also has had a 3‐day history of progressive shortness of breath; he now has shortness of breath at rest. He was evaluated in an urgent care center 1 day ago and was prescribed azithromycin, but his symptoms have worsened. He is using accessory muscles of respiration. He is diaphoretic and is able to give only one- to two-word answers to questions. Vital signs are temperature 39.1°C (102.3°F), pulse 138/min, respirations 40/min, and blood pressure 103/56 mm Hg. Pulse oximetry on room air shows an oxygen saturation of 80%, and 100% oxygen is administered via nonrebreather face mask. Skin is mottled and there is cyanosis of the fingers and toes. Pupils measure 4 mm in diameter and are reactive to light. Mucous membranes are dry. Auscultation of the lungs discloses bilateral crackles. The remainder of the physical examination discloses no abnormalities. Laboratory studies are ordered; results of arterial blood gas analysis on 100% oxygen via nonrebreather face mask are shown: PO2: 50 mm Hg PCO2: 44 mm Hg pH: 7.34 Oxygen saturation: 85% Chest x-ray shows bilateral infiltrates with a normal cardiac silhouette and no effusions. Which of the following is the most likely underlying cause of this patient's condition? (A) Abnormalities of diffusion of oxygen (B) Hypoventilation (C) Obstructive lung disease (D) Ventilation-perfusion mismatch and shunt

**AI_D:** RESTATING THE QUESTION: This patient is experiencing severe symptoms consistent with acute respiratory insufficiency, despite having been prescribed antibiotics for what appears to have been initially diagnosed as a simple respiratory infection. The examination reveals an increased respiratory rate, the utilization of accessory muscles for breathing, and reduced oxygen saturation even when administered at 100% via a mask. The question is asking for the most likely underlying pathophysiological cause of this presented critical condition out of the options provided, specifically looking at disturbances in gas exchange within the lungs. PLANNING: Several possibilities must be considered in this context, including abnormalities of oxygen diffusion, hypoventilation (reduced rate or depth of breathing), obstructive lung disease, and ventilation-perfusion mismatch or shunt. Already, we know that this patient's disease process is acutely severe, resistant to a commonly used antibiotic drug, and associated with signs of systemic inflammation and oxygen deprivation, which likely rules out simple hypoventilation or obstructive lung disorder as being too limited in scope. Analyzing the effects of each of these possible pathologies on the patient's respiratory and systemic condition should help identify the most likely underlying cause. CLINICAL REASONING: The progressive shortness of breath, use of accessory muscles, high respiratory rate, and severe hypoxemia (low blood oxygen level) despite supplemental oxygen suggest a process causing severe impairment of gas exchange. This alone is consistent with abnormalities of oxygen diffusion, ventilation-perfusion mismatch, or a shunt. The arterial blood gas (ABG) results with a normal PCO2 (eliminating hypoventilation) but low PO2 and oxygen saturation, despite high-flow oxygen supplementation, suggests something other than a simple diffusion problem as the primary pathology. This could be a ventilation-perfusion mismatch or a shunt. CONCLUDING: When we consider the chest X-ray showing bilateral infiltrates without any cardomegaly or effusions, this would imply an infectious or inflammatory process involving the lung parenchyma rather than a cardiac cause. Bacterial pneumonia would be consistent with all of these findings, which could cause the congestion of pulmonary capillaries, thereby disturbing the ventilation-perfusion balance predominantly through the development of a shunt. RESPONSE: So, the most likely underlying cause of this patient's condition is (D) Ventilation-perfusion mismatch and shunt. This explanation supports the failure of antibiotic treatment, systemic symptoms, severe oxygen deprivation, and evidence of lung involvement seen in the patient.

**AI_B:** RESTATING THE QUESTION: The question asks about the most probable underlying cause in the present clinical situation of a 22-year-old male presenting with a number of respiratory symptoms, including productive cough with green sputum, fever, chills, rigors, progressive and now resting shortness of breath. These symptoms signal a likely infectious process. He was initially treated with azithromycin but his condition has worsened. The clinical examination findings and laboratory results suggest severe hypoxemia (low oxygen in the blood) despite oxygen therapy, possibly indicating a serious condition affecting the lungs. The options provided include abnormalities of oxygen diffusion, hypoventilation, obstructive lung disease, and ventilation-perfusion mismatch/shunt. PLANNING: The main assumption here is that the cause of his condition is related to the pathology of gas exchange in the lungs given his severe hypoxemia in spite of oxygen supplementation. This could be due to abnormal diffusing capacity of the lungs, hypoventilation (resulting in a buildup of CO2 and insufficient O2), obstruction of the airways, or a ventilation-perfusion mismatch/shunting where areas of the lungs are perfused but not adequately ventilated. CLINICAL REASONING: Let's evaluate these possibilities. Firstly, abnormalities of oxygen diffusion typically show an improvement in oxygen saturation when the patient is administered 100% oxygen, but our patient's saturation remains low at 85%. Secondly, hypoventilation typically causes an increase in PCO2 levels due to inadequate exhalation which isn't the case here. Our patient's PCO2 is within a normal range. Thirdly, obstructive lung disease generally presents with chronic symptoms and airway obstruction, which also doesn't match his acute symptomology. Lastly, ventilation-perfusion mismatch and shunt, describes a situation where there is airflow to an area of the lung but no blood flow, or vice versa. This leads to decreased oxygen levels in the blood, which cannot be fully corrected with supplemental oxygen. This is because the lungs are not able to exhale sufficient carbon dioxide or the blood cannot access the well-ventilated areas. CONCLUDING: From the analysis above, it appears that our assumptions lean towards ventilation-perfusion mismatch/shunt as the likely cause of this patient's symptoms and lab findings due to the fact that his condition failed to improve even with high concentrations of supplemental oxygen which is typically seen in shunt situations. RESPONSE: The most likely underlying cause of this patient's condition is (D) Ventilation-perfusion mismatch and shunt.

**AI_C:** RESTATING THE QUESTION: The question presents a case of a 22-year-old male patient who has developed severe respiratory distress along with other symptoms such as fever, productive cough, and cyanosis. Despite being on maximum oxygen supplementation, the patient remains severely hypoxemic. This detail, combined with the presence of bilateral infiltrates on chest x-ray, the rapid course of the disease and the poor response to azithromycin, paint a picture of a severe bilateral lung disease. The question, therefore, is asking us to identify the most likely physiological cause for the patient's pulmonary complications based on the information given. PLANNING: From the information given, we can hypothesize four possible causes that could produce the currently observed abnormalities: (A) Abnormalities of diffusion of oxygen, (B) Hypoventilation, (C) Obstructive lung disease, or (D) Ventilation-perfusion mismatch and shunt. CLINICAL REASONING: We can first rule out hypothesis ‘B’, hypoventilation, because the patient's PCO2 is normal. If hypoventilation was the cause of the patient’s hypoxemia, we would expect an increase in PCO2 due to inadequate expiration of carbon dioxide. The clinical signs like increase in respiratory rate, use of accessory muscle and dry mucus membrane do not support hypoventilation either, these signs indicate high effort to increase ventilation instead. Abnormalities of diffusion (option A), seen mainly in diseases that thicken the alveolar-capillary interface like interstitial lung disease, are unlikely in a young male with an acute illness. Furthermore, his severe hypoxemia refractory to 100% supplemental oxygen is more consistent with shunt physiology, in which a ventilation-perfusion mismatch results in poorly oxygenated blood bypassing lung parenchyma, rather than a diffusion abnormality. Obstructive lung disease (option C) may present with tachypnea, but generally does not cause severe hypoxemia unless there is also considerable ventilation-perfusion mismatch. Also, such diseases usually manifest over a longer period than seen in this patient’s case, so this diagnosis is also unlikely. This leaves us with option D. Ventilation-perfusion mismatch and shunt. This can occur in conditions such as shock or certain lung diseases that affect the ability of the alveoli to adequately oxygenate the blood. This appears to fit best with the patient's symptoms. CONCLUDING: Therefore, it is likely that the patient is suffering from a severe lung disease possibly leading to acute respiratory distress syndrome (ARDS). This condition typically presents with severe hypoxemia, increased work of breathing manifested as tachypnea and use of accessory muscles, and bilateral lung infiltrates seen on chest imaging due to fluid-filled, collapsed alveoli. This fits with the presented case and provides the most plausible explanation for the patient's symptoms. RESPONSE: The underlying cause of this patient's condition is most likely (D) Ventilation-perfusion mismatch and shunt.

**AI_E:** (1) RESTATING THE QUESTION: The patient is a 22-year-old man who is presenting with symptoms consistent with a severe respiratory disorder, which include fever, chills, a productive cough with green sputum, shortness of breath at rest, use of accessory muscles for respiration, diaphoresis, cyanosis of fingers and toes, dry mucous membranes, and bilateral crackles upon lung auscultation. Despite an initial treatment with azithromycin at an urgent care center, the symptoms have worsened. His current condition continues to deteriorate even with administration of 100% oxygen via a nonrebreather face mask. His blood gas analysis reveals a low oxygen level (PO2: 50 mm Hg), a normal-to-slight high carbon dioxide level (PCO2: 44 mm Hg) and relatively low blood pH (7.34), indicating a state of mild acidosis. Chest x-ray shows bilateral infiltrates, a normal cardiac silhouette and no effusions. The question now is the actual underlying cause of the patient's severe respiratory condition from the given choices: abnormal diffusion of oxygen, hypoventilation, obstructive lung disease, or ventilation-perfusion mismatch and shunt. (2) PLANNING: To understand the possible cause of the patient's condition, we may need to evaluate each choice provided. Diffusion abnormalities typically result in low PaO2 while oxygen administration significantly improves PaO2. Hypoventilation usually leads to hypercapnia, but in this case, the patient's PCO2 is normal. Obstructive lung diseases such as COPD or asthma are possibilities, but they usually involve a chronic process and present with different symptoms and physical examination findings than we have here. Lastly, a ventilation-perfusion (V/Q) mismatch or shunt is another possible cause. (3) CLINICAL REASONING: The given history and physical exam findings, along with the patient's worsening condition despite the administration of 100% oxygen, seem not to coincide with a simple case of hypoventilation or obstructive lung disease. Hypoventilation would have resulted not only in lower oxygen levels, but also elevated levels of carbon dioxide, which is not the case with this patient given his normal PCO2. Obstructive lung disease could be suspected, but the acute nature and severity of this case, as well as the lack of classic obstructive disease characteristics, make it a less likely possibility. Regarding diffusion abnormalities, they generally cause a reduced PaO2 but when inhaled oxygen concentration is increased to 100%, we would expect a significant rise in PaO2, which is not seen here. Considering a ventilation-perfusion (V/Q) mismatch, this is when there is an imbalance between alveolar ventilation and pulmonary capillary perfusion. A type of V/Q mismatch - a shunt - happens when parts of the lung are perfused but not ventilated. In situations like this, administering oxygen does not improve oxygenation as much, as is seen with our patient. Therefore, upon clinical reasoning, the abnormalities of this patient's condition point towards a severe form of pneumonia that has possibly formed a consolidation, resulting in a large ventilation-perfusion mismatch and possibly a shunt. (4) CONCLUDING: Given the patient's young age, absence of risk factors for chronic diseases, and acute symptom onset, the prevailing infection symptoms, combined with lab results showing infiltrates on chest x-ray and diminished response to oxygen administration, this leads us to conclude that there's a practically significant likelihood for a condition that can cause a ventilation-perfusion mismatch and shunt, likely due to severe pneumonia. (5) RESPONSE: Thus, the most likely underlying cause of this patient's condition is a ventilation-perfusion mismatch and shunt (D), likely due to severe pneumonia leading to shunting and significantly reduced gas exchange, and that is why oxygen administration wasn't able to improve his oxygenation.

**AI_A:** RESTATING THE QUESTION: A 22-year-old male patient is experiencing severe respiratory distress, with symptoms including rapid pulse, high fever, shortness of breath, low oxygen saturation, productive cough and bilateral crackles on lung auscultation. His condition has worsened despite treatment with azithromycin, and he presents signs of cyanosis. Lab tests reveal decreased PO2 and oxygen saturation levels even after oxygen administration. Chest x-ray displays bilateral lung infiltrates. The query is about the primary causative factor for this man's respiratory ailment and the options include: (A) Oxygen diffusion disturbances, (B) Hypoventilation, (C) Obstruction of the lungs, and (D) A discrepancy in ventilation and perfusion (V/Q mismatch) along with a shunt. PLANNING: Considering the data, the assumption that the patient possibly has a serious respiratory infection, such as pneumonia, given his fever, productive cough, and bilateral crackles, is reasonable. However, for careful clinical decision-making, each option needs to be evaluated: (A) Abnormalities of diffusion of oxygen - Could the patient's condition be due to an inability of the oxygen to diffuse properly through the lung membrane? (B) Hypoventilation - Is his body failing to eliminate enough carbon dioxide, causing a buildup and leading to respiratory acidosis? (C) Obstructive lung disease - Could there be a persistent blockage restricting airflow? (D) Ventilation-perfusion mismatch and shunt - Is there an imbalance in the air and blood flow in the lungs, causing less effective oxygenation? CLINICAL REASONING: (A) The abnormally low PO2 and oxygen saturation even after oxygen administration does suggest a potential problem with the oxygen’s ability to diffuse from the alveoli into the bloodstream. However, the other clinical features seen in this patient, like sputum production and fever, point towards an infection at play. (B) Hypoventilation typically leads to hypercapnia (elevated PCO2), which escalates to respiratory acidosis. The arterial blood gas results in this case show a normal PCO2 and a pH close to the normal value, indicating that ventilation is maintained. (C) Obstructive lung diseases (like asthma, emphysema) commonly present with wheezes and dyspnea, but not with fever or sputum. The bilateral infiltrates on chest x-ray mainly indicate an infectious or inflammatory etiology rather than an obstructive pathology. (D) A ventilation-perfusion mismatch implies an uneven distribution of ventilation and blood flow (perfusion) in the lungs. A shunt refers to blood bypassing the alveoli without gas exchange. In severe infections, inflammation and consolidation shift blood flow away from involved areas, leading to V/Q mismatch and/or shunt. This seems to align well with the clinical presentation and the failure to significantly improve PO2 with supplemental oxygen, indicating a large shunt effect. CONCLUDING: Careful analysis of the clinical picture, coupled with understanding the pathophysiology of lung conditions, indicates that while each option might contribute to the overall picture, the Ventilation-perfusion mismatch and shunt is the most likely primary cause driving the hypoxemia in the context of a severe pneumonia. RESPONSE: Thus, the most probable underlying cause of this patient's condition is (D) Ventilation-perfusion mismatch and shunt in the context of severe pneumonia.

**Facilitator** The question presents a case of a 22-year-old male patient who is experiencing severe respiratory distress, with symptoms including rapid pulse, high fever, shortness of breath, low oxygen saturation, productive cough, and bilateral crackles on lung auscultation. Despite treatment with azithromycin, his condition has worsened, and he presents signs of cyanosis. Lab tests reveal decreased PO2 and oxygen saturation levels even after oxygen administration. Chest x-ray displays bilateral lung infiltrates. The query is about the primary causative factor for this man's respiratory ailment and the options include: (A) Oxygen diffusion disturbances, (B) Hypoventilation, (C) Obstruction of the lungs, and (D) A discrepancy in ventilation and perfusion (V/Q mismatch) along with a shunt. Upon careful analysis of the clinical picture and understanding the pathophysiology of lung conditions, it is clear that while each option might contribute to the overall picture, the Ventilation-perfusion mismatch and shunt is the most likely primary cause driving the hypoxemia in the context of a severe pneumonia. This conclusion is based on the patient's severe hypoxemia refractory to 100% supplemental oxygen, which is more consistent with shunt physiology, in which a ventilation-perfusion mismatch results in poorly oxygenated blood bypassing lung parenchyma, rather than a diffusion abnormality. The patient's condition failed to improve even with high concentrations of supplemental oxygen, which is typically seen in shunt situations. The chest X-ray showing bilateral infiltrates without any cardomegaly or effusions implies an infectious or inflammatory process involving the lung parenchyma rather than a cardiac cause. Bacterial pneumonia would be consistent with all of these findings, which could cause the congestion of pulmonary capillaries, thereby disturbing the ventilation-perfusion balance predominantly through the development of a shunt. Therefore, the consensus statement is that the most likely underlying cause of this patient's condition is (D) Ventilation-perfusion mismatch and shunt in the context of severe pneumonia. This explanation supports the failure of antibiotic treatment, systemic symptoms, severe oxygen deprivation, and evidence of lung involvement seen in the patient.
